# Supplementary material for: Phenotype prediction using biologically interpretable neural networks on multi-cohort multi-omics data
Source: NPJ Syst Biol Appl. 2024 Aug 2;10:81. doi: 10.1038/s41540-024-00405-w (PMC11297229; doi:10.1038/s41540-024-00405-w)
Supplement: Supplementary file 1 — Supplementary Material [file 41540_2024_405_MOESM1_ESM.pdf]

Phenotype prediction using biologically interpretable neural networks on multi-cohort multi-omics data

Table of Contents

SUPPLEMENTARY NOTE 2
1. L1 REGULARIZATION 2
2. BASELINE NEURAL NETWORK 2
3. PCA ON THE ACTIVATIONS OF THE NEURONS 2
SUPPLEMENTARY TABLES 3
1. PERFORMANCE OF REGULARIZED LINEAR AND LOGISTIC REGRESSION 3
2. PERFORMANCE OF BASELINE NEURAL NETWORKS 3
3. HYPERPARAMETERS 4
4. SMOKING STATUS PREDICTION 6
5. AGE PREDICTION 8
7. LDL-LEVEL PREDICTION 10
SUPPLEMENTARY FIGURES 11
1. COHORT-WISE CROSS-VALIDATION 11
2. PATHWAY NETWORK: NUMBER OF GENES PER PATHWAY 11
3. SMOKING: PATHWAY IMPORTANCE 12
4. SMOKING STATUS: OMIC-SPECIFIC INFORMATION 13
5. AGE PREDICTION 15
6. AGE: SEX SPECIFIC EFFECTS 16
7. AGE: OMIC SPECIFIC EFFECTS 19
8. AGE: PATHWAY IMPORTANCE 21
9. LDL PREDICTION 22

Supplementary Note

1. L1 regularization

The L1 penalty is implemented using the Tensorflow kernel regularizer method. For each layer with the L1 penalty the following term is added to the loss function.

$$Loss = MSE \ or \ BCE \ + r\sum_{i=1}^{N}|w_i|$$

With r as a hyperparameter balancing the L1 term and with N as the number of weights in that layer. For regression tasks, the binary cross-entropy (*BCE*) loss was used while for regression tasks the mean squared error (*MSE*) was utilized in the loss function. A custom L1 regularization function was written for the omic-specific L1 penalty. In this function, only the weights of a specific omic are penalized.

For each layer except for the output layer, a default of $r = 1\times10^{-8}$ was chosen. The L1 penalty hyperparameter for the output layer was optimized per cohort and task. We added this stronger L1 penalty to improve interpretation for the output layer, since this is a fully connected neuron unguided by prior biological knowledge.

2. Baseline neural network

To compare the performance of visible neural networks to regular neural networks, we trained a three-layer deep neural network using the same cohort-wise cross-validation setup. A three layer fully connected network (100, 50, 1 nodes) resulted in more than 48 million trainable parameters. This fully connected network did not converge satisfactory and therefore we proceeded with the LocallyConnected layer. The resulting baseline networks consists thus of a LocallyConnected layer with a window size of 20 and stride 10, followed by a fully connected layers with 50 neurons and a fully connected layer with a single output neuron. For the multi-omics data, the methylation and gene expression data were first concatenated before feeding it to the network. We optimized the learning rate and architectural choices (type and number of layers) on the validation set.

The LocallyConnected1D layer has been successfully used in various application in genomics[2–4]. This layers can be seen as a hybrid between convolutional and fully-connected layers[5], using a number of filters with a certain window size that are repeated with a specific stride. Unlike in CNNs, where filters slide over the input, the filters in LocallyConnected are static, akin to those in fully connected networks.

The documentation of this layer for the used Tensorflow version (v2.2) can be found here: https://www.tensorflow.org/versions/r2.2/api_docs/python/tf/keras/layers/LocallyConnected1D

3. PCA on the activations of the neurons

For each patient all the activation values of the neural network are gathered and concatenated. The activation value is the value obtained after applying the activation function (ReLu, tanh, or sigmoid). The resulting matrix, with dimensions equal to the number of individuals in the training set and the number of

neurons in the whole network, is used for principal component analysis. Scikit-learn's PCA function sklearn.decomposition.PCA (v1.4.2) has been used to do the principal component analysis.

A neural network can lean multiple patterns to predict a single outcome. The contribution scores based on the weights provide a global interpretation that fails to show this individual difference. The weights represent an average over the whole population. Individually, the neural network could use different patterns. A PCA on the activation can show the different activation patterns for the nodes, which can be leveraged to find groups of individuals that have a different underlying mechanism that still leads to the same outcome.

Supplementary Tables

1. Performance of regularized linear and logistic regression

**Validation set**					
		**Rotterdam Study**	**LifeLines**	**Leiden Longevity Study**	**Netherlands Twin Register**
**Smoking [AUC]**	ME	0.71	0.71	0.69	0.75
	GE	0.80	0.83	0.80	0.82
**Age [expl. var.]**	ME	0.97	0.96	0.96	0.95
	GE	0.83	0.83	0.82	0.81
**LDL [expl. var.]**	ME	0.08	0.07	0.06	0.00
	GE	0.13	0.13	0.11	0.04
**Test set**					
		**Rotterdam Study**	**LifeLines**	**Leiden Longevity Study**	**Netherlands Twin Register**
**Smoking [AUC]**	ME	0.77	0.77	0.70	0.73
	GE	0.93	0.80	0.88	0.77
**Age [expl. var.]**	ME	0.76	0.93	0.67	0.60
	GE	0.63	-0.20	-0.25	-0.31
**LDL [expl. var.]**	ME	0.08	0.07	0.00	0.00
	GE	-0.01	0.03	-0.00	-0.12

Supplementary Table 1. Performance of out-of-the-box scikit-learn implementations. LogisticRegression with an L1 penalty for predicting smoking status and LASSO for LDL and Age prediction.

2. Performance of baseline neural networks

**Phenotype**	**Network type**	**AUC validation cohorts**	**AUC test cohort**	**Mean test AUC over all cohorts**

		RS*	LL*	LLS*	NTR*	RS	LL	LLS	NTR	
**Smoking**	**ME+GE**	0.88 (0.87 - 0.88)	0.91 (0.91 - 0.91)	0.90 (0.90 - 0.90)	0.90 (0.89 - 0.90)	0.96 (0.96 - 0.96)	0.92 (0.92 - 0.92)	0.94 (0.94 - 0.94)	0.85 (0.84 - 0.85)	**0.92 (0.84 - 1.00)**
	**ME**	0.88 (0.87 - 0.88)	0.87 (0.87 - 0.88)	0.91 (0.91 - 0.92)	0.89 (0.88 - 0.89)	0.95 (0.95 - 0.95)	0.90 (0.90 - 0.91)	0.89 (0.89 - 0.90)	0.85 (0.85 - 0.86)	**0.90 (0.84 - 0.96)**
	**GE**	0.84 (0.83 - 0.85)	0.89 (0.89 - 0.90)	0.88 (0.87 - 0.89)	0.88 (0.88 - 0.89)	0.94 (0.94 - 0.95)	0.90 (0.89 - 0.91)	0.92 (0.91 - 0.93)	0.81 (0.79 - 0.82)	**0.90 (0.82 - 0.99)**
**Phenotype**	**Network type**	**RMSE validation cohorts**				**RMSE test cohorts**				**Mean test RSME over all cohorts**
		**RS***	**LL***	**LLS***	**NTR***	**RS**	**LL**	**LLS**	**NTR**	
**Age**	**ME+GE**	3.63 (3.42 - 3.85)	4.06 (3.71 - 4.40	3.56 (3.44 - 3.68)	3.62 (3.54 - 3.70)	5.60 (5.34 - 5.85)	6.43 (5.52 - 7.35)	4.34 (4.14 - 4.55)	6.22 (5.76 - 6.68)	**5.90 (4.59 - 7.21)**
	**ME**	3.03 (2.93-3.04)	3.39 (3.25 - 3.53)	2.96 (2.89 - 3.04)	3.26 (3.16 - 3.37)	5.32 (5.16 - 5.48)	4.80 (4.51 - 5.10)	3.18 (3.12 - 3.25)	4.06 (3.65 - 4.46)	**4.10 (2.73 - 5.46)**
	**GE**	6.66 (6.58 - 6.74)	6.57 (6.44 - 6.70)	6.32 (6.21 - 6.44)	6.07 (6.00 - 6.14)	9.77 (9.35 - 10.19)	13.79 (13.22 - 14.36)	8.66 (7.85 - 9.48)	17.33 (16.4 - 18.26)	**11.32 (5.60 - 17.05)**
**LDL**	**ME+GE**	0.88 (0.87 - 0.88)	0.89 (0.88 - 0.89)	0.86 (0.85 - 0.86)	0.90 (0.89 - 0.90)	0.93 (0.92 - 0.93)	0.92 (0.91 - 0.92)	0.95 (0.94 - 0.95)	1.00 (0.98 - 1.02)	**0.95 (0.86 - 1.04)**
	**ME**	0.89 (0.89 - 0.89)	0.90 (0.90 - 0.91)	0.91 (0.9 - 0.92)	0.92 (0.92 - 0.92)	0.95 (0.94 - 0.95)	0.90 (0.90 - 0.91)	0.95 (0.94 - 0.95)	0.98 (0.97 - 0.98)	**0.94 (0.89 - 0.99)**
	**GE**	0.96 (0.94 - 0.97)	0.96 (0.95 - 0.98)	1.16 (0.63 - 1.69)	0.96 (0.94 - 0.98)	0.99 (0.98 - 1.01)	0.98 (0.97 - 0.99)	1.27 (0.71 - 1.82)	1.25 (1.17 - 1.34)	**1.09 (0.86 - 1.32)**

*Supplementary Table 2. Baseline, three-layer deep neural network (locallyconnected1D layer followed by two dense layers), performance for cohort-wise cross validation, mean with 95% confidence interval over 10 runs. The area under the curve is reported for the classification task (smoking status prediction) and the root mean squared error (RMSE) for the regression tasks, predicting age and LDL levels. ME; Methylation, GE; Gene expression, ME+GE, both methylation and gene expression as an input for the neural network. RS; Rotterdam study, LL; LifeLines, NTR; Netherlands Twin Register, LLS; Leiden Longevity Study. See Supplementary Table 1 for the performance of out-of-the-box scikit-learn implementations for each omic. *The name of the test cohort is used to denote the fold. Thus, for the first fold, RS, was used for testing and LL+LLS+NTR were used for training and validation (75% training, 25% validation).*

3. Hyperparameters

			ME+GE	ME	GE	Deep dense

	Fold (by test set)	Abbreviation	L1	LR	L1	LR	L1	LR	L1	LR
Smoking	Rotterdam Study	RS	0.001	0.01	0.001	0.01	0.0001	0.001	0.001	0.001
	LifeLines	LL	0.001	0.01	0.001	0.01	0.0001	0.001	0.001	0.001
	Longevity Study	LLS	0.001	0.01	0.001	0.01	0.0001	0.001	0.0001	0.005
	Netherlands Twin Register	NTR	0.001	0.01	0.001	0.01	0.0001	0.001	0.001	0.001
Age	Rotterdam Study	RS	0.01	0.005	0.01	0.01	0.001	0.01	0.01	0.0001
	LifeLines	LL	0.01	0.005	0.01	0.01	0.001	0.01	0.001	0.001
	Longevity Study	LLS	0.01	0.005	0.01	0.01	0.001	0.01	0.001	0.0001
	Netherlands Twin Register	NTR	0.01	0.005	0.01	0.01	0.001	0.01	0.01	0.1
LDL	Rotterdam Study	RS	0	0.01	0.001	0.01	0.0001	0.001	0.001	0.0001
	LifeLines	LL	0	0.01	0.001	0.01	0.0001	0.001	0.01	0.01
	Longevity Study	LLS	0	0.01	0.001	0.01	0.0001	0.001	0.001	0.000001
	Netherlands Twin Register	NTR	0	0.01	0.001	0.01	0.0001	0.001	0.001	0.000001

Supplementary Table 3. Hyperparameters for the best performance in the validation set. Batch size was in all experiments 64, L1 penalty value (L1) was on the kernel regularization penalty, learning rate (LR) was for the ADAM optimizer.

4. Smoking status prediction

Smoking status prediction									
**Network type**	**AUC validation cohorts (excluded test cohort)**				**AUC test cohort (test cohort)**				**Mean over all cohorts**
**-**	**RS**	**LL**	**LLS**	**NTR**	**RS**	**LL**	**LLS**	**NTR**	
**ME+GE**	0.92 (0.90 - 0.94)	0.93 (0.92 - 0.93)	0.91 (0.90 - 0.92)	0.93 (0.91 - 0.94)	0.98 (0.98 - 0.98)	0.92 (0.92 - 0.93)	0.95 (0.95 - 0.96)	0.91 (0.89 - 0.92)	**0.95 (0.90 – 1.00)**
**ME**	0.93 (0.92 - 0.94)	0.94 (0.94 - 0.95)	0.95 (0.94 - 0.95)	0.93 (0.93 - 0.94)	0.97 (0.97 - 0.98)	0.94 (0.93 - 0.94)	0.96 (0.95 - 0.96)	0.95 (0.95 - 0.96)	**0.95 (0.93 - 0.98)**
**GE**	0.83 (0.83 - 0.84)	0.82 (0.82 - 0.82)	0.83 (0.83 - 0.83)	0.87 (0.86 - 0.87)	0.87 (0.87 - 0.88)	0.85 (0.85 - 0.85)	0.87 (0.87 - 0.88)	0.80 (0.80 - 0.80)	**0.85 (0.80 - 0.90)**
**ME+GE (penalized methylation L1 of 0.001)**	0.88 (0.87 - 0.89)	0.88 (0.87 - 0.89)	0.9 (0.9 - 0.91)	0.91 (0.91 - 0.92)	0.95 (0.94 - 0.96)	0.88 (0.88 - 0.89)	0.94 (0.94 - 0.94)	0.86 (0.86 - 0.87)	**0.91 (0.83 - 0.99)**
**ME+GE (penalized gene expression L1 of 0.001)**	0.92 (0.90 - 0.94)	0.92 (0.90 - 0.93)	0.91 (0.89 - 0.93)	0.92 (0.91 - 0.93)	0.98 (0.97 - 0.98)	0.92 (0.91 - 0.93)	0.95 (0.95 - 0.96)	0.89 (0.87 - 0.91)	**0.95 (0.89 - 1.0)**
**Deep (5 layers dense)**	0.83 (0.81 - 0.85)	0.86 (0.85 - 0.87)	0.84 (0.83 - 0.85)	0.89 (0.88 - 0.90)	0.95 (0.94 - 0.95)	0.90 (0.89 - 0.90)	0.89 (0.87 - 0.90)	0.84 (0.82 - 0.86)	**0.91 (0.85 - 0.96)**
**Pathway**	0.83 (0.82 - 0.84)	0.83 (0.82 - 0.84)	0.76 (0.75 - 0.78)	0.85 (0.84 - 0.85)	0.89 (0.89 - 0.9)	0.84 (0.84 - 0.85)	0.81 (0.78 - 0.84)	0.8 (0.78 - 0.81)	**0.84 (0.77 - 0.91)**

Supplementary Table 4. Results for the base network and variations to the base network. Area under the curve for smoking status prediction for each cohort in a cohort-wise cross validation, mean with 95% confidence interval over 10 runs.

Gene	Mean percentage fold 1	Mean percentage fold 2	Mean percentage fold 3	Mean percentage fold 4	Mean all folds
GPR15	4.64	2.73	3.24	4.44	3.76
AHRR	3.41	2.50	0.00	5.60	2.88
LRRN3	2.40	1.93	2.10	2.85	2.32
SEMA6B	2.02	2.11	2.10	2.86	2.27
P2RY6	2.27	2.09	2.02	1.61	2.00
CDKN1C	1.74	1.42	0.98	2.27	1.60
KCNQ1	1.07	1.69	1.12	1.73	1.40
PID1	1.00	1.20	1.08	1.24	1.13
CLEC10A	1.00	0.98	1.07	1.30	1.09

Supplementary Table 5: Genes with contributions higher than 1% of the total weight for smoking prediction for three out of the four folds.

5. Age prediction

6. Gene	Mean percentage fold 1	Mean percentage fold 2	Mean percentage fold 3	Mean percentage fold 4	Mean all folds
COL11A2	0.57	0.55	0.34	0.34	0.61
AFAP1	0.85	0.59	0.53	0.53	0.60
OTUD7A	0.37	0.32	0.63	0.63	0.55
PTPRN2	0.28	0.80	0.43	0.43	0.51
ADARB2	0.49	0.59	0.40	0.40	0.48
CD34	0.20	0.42	0.64	0.64	0.45
AGAP1	0.43	0.44	0.52	0.52	0.44
IRS2	0.47	0.24	0.42	0.42	0.43
DPYSL4	0.37	0.19	0.75	0.75	0.42
CACNA1I	0.59	0.37	0.35	0.35	0.41
DNAJB6	0.59	0.39	0.21	0.21	0.40
FBXO31	0.46	0.44	0.17	0.17	0.38
GRM2	0.33	0.36	0.70	0.70	0.35
PPP2R2D	0.00	0.38	0.39	0.39	0.34
MGMT	0.08	0.33	0.31	0.31	0.29
CHMP6	0.14	0.31	0.32	0.32	0.29

Supplementary Table 6. Genes with contributions higher than 1% of the total weight for biological age prediction for three out of the four folds.

Age Prediction									
	R^2 validation cohorts (excluding named cohort)				R^2 (test cohort)				Mean over all test cohorts
	RS	LL	LLS	NTR	RS	LL	LLS	NTR	
**ME+GE**	0.94 (0.93 - 0.94)	0.94 (0.94 - 0.94)	0.95 (0.95 - 0.95)	0.90 (0.89 - 0.91)	0.40 (0.37 - 0.43)	0.88 (0.87 - 0.88)	0.61 (0.60 - 0.63)	0.91 (0.90 - 0.92)	**0.72 (0.36 - 1.07)**

**ME**	0.23 (0.10 - 0.36)	0.81 (0.76 - 0.86)	0.32 (0.16 - 0.48)	0.35 (0.23 - 0.48)	-0.30 (-0.87 - 0.27)	0.75 (0.69 - 0.81)	-0.15 (-0.52 - 0.23)	0.31 (0.24 - 0.38)	**-0.31 (-2.5 - 1.88)**
**GE**	0.81 (0.81 - 0.81)	0.85 (0.85 - 0.85)	0.87 (0.87 - 0.87)	0.77 (0.77 - 0.78)	-0.08 (-0.09 - -.06)	0.64 (0.64 - 0.64)	0.09 (0.09 - 0.09)	0.55 (0.54 - 0.56)	**0.30 (-0.26 - 0.86)**
**ME+GE (L1 ME)**	0.93 (0.93 - 0.94)	0.93 (0.93 - 0.94)	0.95 (0.94 - 0.95)	0.89 (0.88 - 0.89)	0.39 (0.34 - 0.44)	0.87 (0.87 - 0.88)	0.59 (0.57 - 0.61)	0.90 (0.89 - 0.9)	**0.69 (0.29 - 1.08)**
**ME+GE (L1 GE)**	0.94 (0.93 - 0.94)	0.93 (0.93 - 0.94)	0.95 (0.95 - 0.95)	0.89 (0.89 - 0.90)	0.40 (0.35 - 0.44)	0.87 (0.87 - 0.88)	0.60 (0.59 - 0.62)	0.90 (0.89 - 0.91)	**0.73 (0.42 - 1.03)**
**Pathway**	0.66 (0.59 - 0.74)	0.82 (0.78 - 0.86)	0.82 (0.78 - 0.85)	0.53 (0.47 - 0.58)	-0.62 (-.76 - -.48)	0.52 (0.43 - 0.61)	-0.26 (-.47 - -.05)	0.28 (0.15 - 0.41)	**0.07 (-0.82 - 0.68)**
**ME+GE +sex**	0.56 (0.43 - 0.69)	0.81 (0.77 - 0.85)	0.83 (0.77 - 0.89)	0.53 (0.41 - 0.64)	-0.42 (-.63 - -.20)	0.52 (0.38 - 0.65)	-0.2 (-0.47 - 0.07)	0.26 (0.11 - 0.41)	**0.09 (-0.54 - 0.72))**
**ME+GE+ sex per gene**	0.01 (-1.11 - 1.13)	0.84 (0.71 - 0.97)	0.67 (0.47 - 0.87)	0.26 (-0.98 - 1.5)	-4.22 (-10.57 - 2.14)	0.75 (0.67 - 0.84)	-0.48 (-1.33 - 0.37)	0.58 (0.09 - 1.07)	**0.37 (-0.8 - 1.54)**
**Deeper (3 densely connected)**	0.81 (0.79 - 0.82)	0.84 (0.84 - 0.85)	0.87 (0.86 - 0.87)	0.76 (0.74 - 0.77)	-0.18 (-0.32 - -.05)	0.56 (0.54 - 0.59)	-0.27 (-0.33 - -.21)	0.58 (0.56 - 0.61)	**0.14 (-0.63 - 0.91)**

Supplementary Table 7. Results for the base network and variations to the base network. Age prediction performance in explained variance (R^2) for each cohort in a cohort-wise cross validation, mean over 10 runs with 95% confidence interval.

7. LDL-level prediction

LDL-level prediction									
	R^2<br>validation cohorts<br>(excluding named cohort)				R^2<br>(test cohort)				Mean<br>over all cohorts
	RS	LL	LLS	NTR	RS	LL	LLS	NTR	
**ME+GE**	0.14<br>(0.13 - 0.15)	0.13<br>(0.12 - 0.14)	0.17<br>(0.16 - 0.18)	0.05<br>(0.05 - 0.05)	0.01<br>(-.00 - 0.01)	0.07<br>(0.05 - 0.08)	-0.01<br>(-0.02 - 0.0)	0.01<br>(0.01 - 0.02)	**0.02**<br>**(-0.04 - 0.08)**
**ME**	-0.4<br>(-0.61 - -0.18)	-0.3<br>(-0.45 - -0.14)	-0.32<br>(-.56 - -0.08)	-0.38<br>(-.54 - -0.23)	-0.56<br>(-.85 - -.28)	-0.42<br>(-.65 - -.19)	-0.31<br>(-.55 - -.06)	-0.38<br>(-.54 - -.21)	**-0.56**<br>**(-1.38 - 0.26)**
**GE**	0.13<br>(0.12 - 0.13)	0.12<br>(0.12 - 0.13)	0.16<br>(0.16 - 0.17)	0.05<br>(0.05 - 0.06)	-0.01<br>(-.01 - -.00)	0.04<br>(0.04 - 0.05)	-0.01<br>(-0.01 - -.00)	-.06<br>(-.08 - - .04)	**-0.02**<br>**(-0.09 - 0.06)**
**ME+GE**<br>**(L1 ME)**	0.12<br>(0.11 - 0.13)	0.11<br>(0.09 - 0.12)	0.14<br>(0.12 - 0.15)	0.05<br>(0.04 - 0.07)	0.00<br>(-0.02 - 0.03)	0.05<br>(0.04 - 0.06)	-0.05<br>(-.07 - -.02)	0.02<br>(-.00 - .03)	**-0.00**<br>**(-0.07 - 0.06)**
**ME+GE**<br>**(L1 GE)**	0.10<br>(0.09 - 0.12)	0.08<br>(0.06 - 0.10)	0.09<br>(0.07 - 0.10)	0.04<br>(0.03 - 0.05)	-0.03<br>(-0.06 - -.01)	0.02<br>(0.01 - 0.03)	-0.01<br>(-0.02 - 0.01)	-0.03<br>(-.05 - -.01)	**-0.01**<br>**(-0.04 - 0.03)**
**Deeper**<br>**(3 densely connected)**	0.11<br>(0.07 - 0.15)	0.01<br>(-.01 - 0.02)	0.12<br>(0.10 - 0.14)	0.04<br>(0.03 - 0.05)	0.0<br>(-.01 - .01)	-0.0<br>(-0.01 - 0.00)	-0.0<br>(-.01 - 0.01)	0.01<br>(0.00 - 0.01)	**-0.00**<br>**(-0.01 - 0.01)**
**Deeper**<br>**(Pathway)**	0.11<br>(0.10 - 0.11)	0.11<br>(0.10 - 0.11)	0.11<br>(0.10 - 0.11)	0.05<br>(0.05 - 0.06)	-0.03<br>(-.04 - -.010)	0.03<br>(0.02 - 0.03)	-0.0<br>(-.01 - 0.00)	-0.01<br>(-.02 - 0.00)	**-0.01**<br>**(-0.04 - 0.02)**
**ME+GE**<br>**+sex**	-0.49<br>(-.93 - -0.06)	0.02<br>(-0.03 - 0.08)	-1.34<br>(-1.66 - -1.01)	-2.22<br>(-2.76 - -1.68)	-0.79<br>(-1.68 - 0.1)	-0.03<br>(-.10 - 0.04)	-1.59<br>(-1.98 - -1.20)	-2.22<br>(-2.81 - -1.62)	**-1.60**<br>**(-3.35 - 0.15)**

Supplementary Table 8. Performance for cohort-wise cross validation for LDL level prediction, mean with 95% CI over 10 runs. Explained variance of 0 is equal to just predicting the mean. Explained variance can be negative when the network overfits and, consequently, has poorer predictive performance than predicting the mean in the test set.

Supplementary Figures

1. Cohort-wise cross-validation

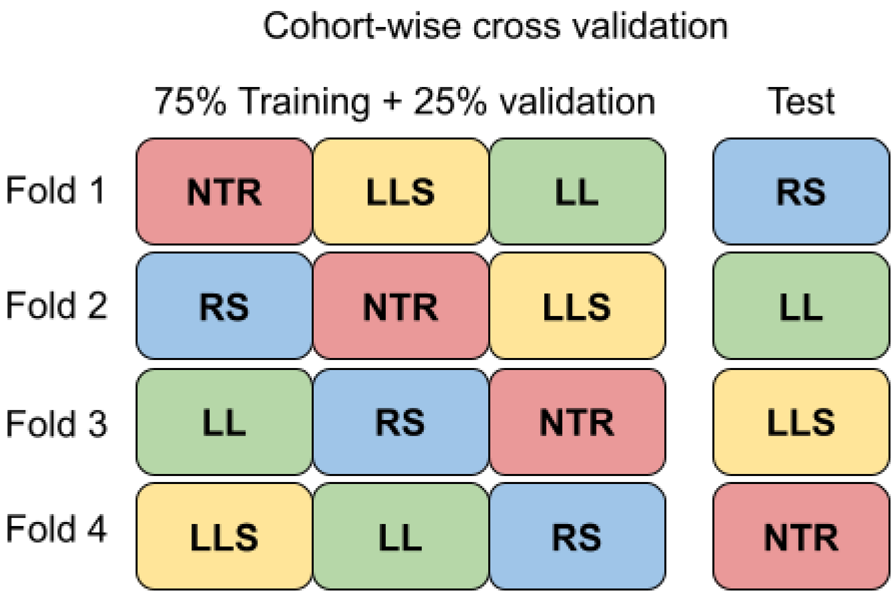

Supplementary Figure 1. Cohort-wise cross-validation. For each fold three cohorts were used to train and validate the hyperparameters of the model (75% training, 25% validation). The remaining, left-out cohort served as an independent test set and the average performance over the test cohorts was reported. The cohort-wise cross validation was done for each phenotype (smoking, LDL and age prediction). Abbreviations: Netherlands Twin Register (NTR), Leiden Longevity Study (LLS), LifeLines (LL), Rotterdam Study (RS).

2. Pathway network: number of genes per pathway

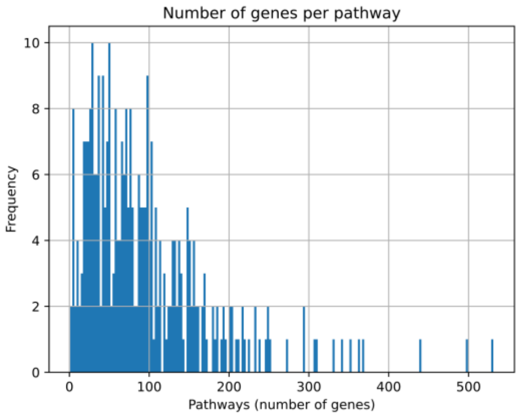

Supplementary Figure 2. Distribution of the number of genes assigned per pathway. The same KEGG pathway annotations were used for each pathway neural network. Each pathway with more than one gene assigned to it was included in the network. There are twelve pathways with less than 10 genes.

3. Smoking: pathway importance

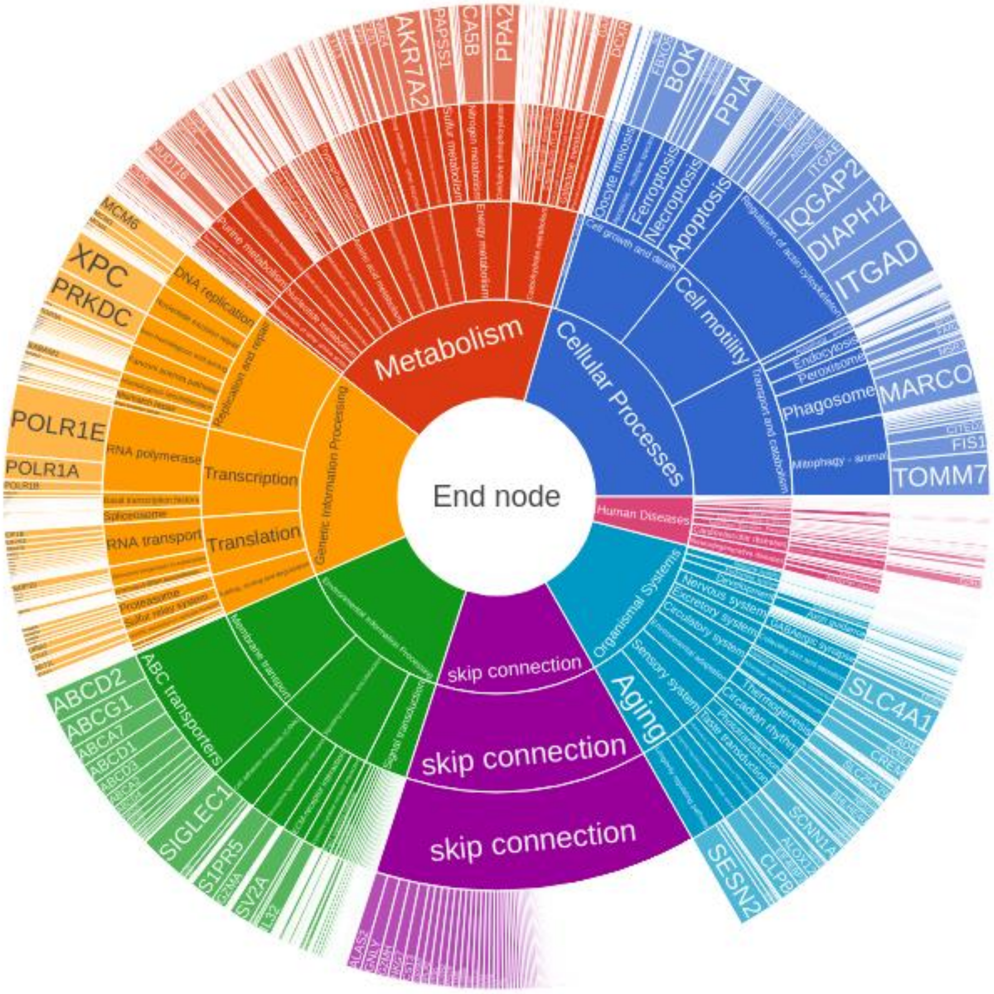

Supplementary Figure 3. Importance of KEGG functional pathways and their corresponding genes for predicting smoking status. Skip connections connect each gene right away to the end node to ensure that each gene is connected to the output.

4. Smoking status: omic-specific information

Simulated data for subtype analysis

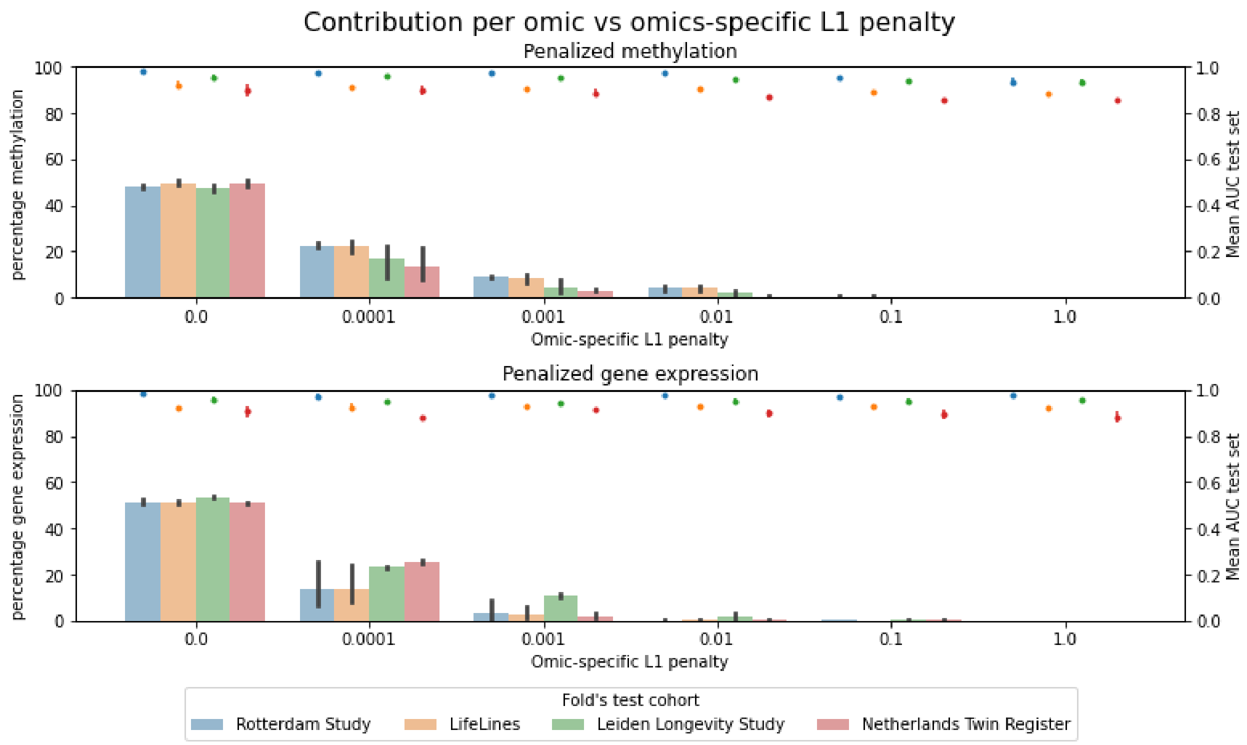

Supplementary Figure 4. Contribution per omic versus omics-specific L1 penalty. The bar plots show the percentage of the total weight of the penalized omics. Additionally, the mean AUC and standard deviation is denoted by the colored points using the right axis.

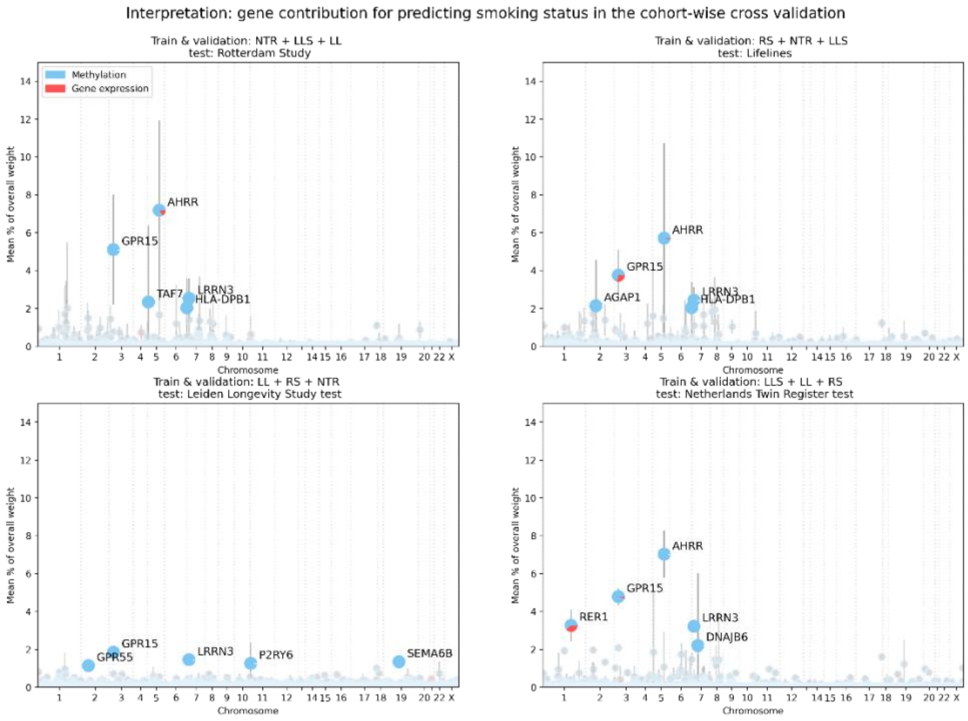

Supplementary Figure 5. Smoking prediction with the ME+GE network penalized for gene expression genes with an omic-specific L1 penalty of 0.001.

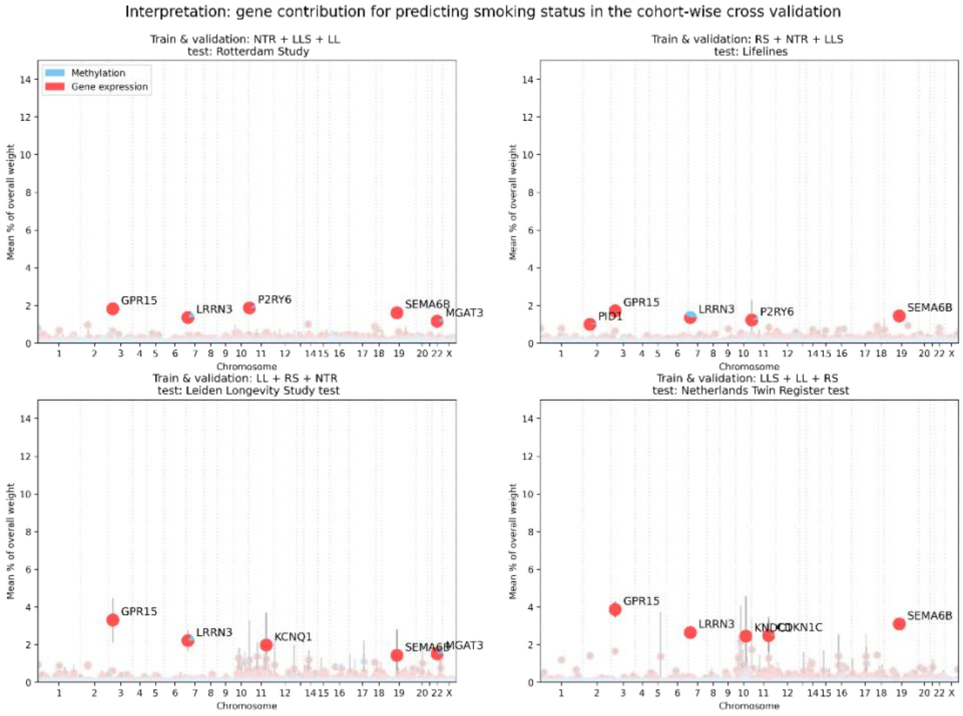

Supplementary Figure 6. Smoking prediction with the ME+GE network penalized for methylation genes with an omic-specific L1 penalty of 0.001.

5. Age prediction

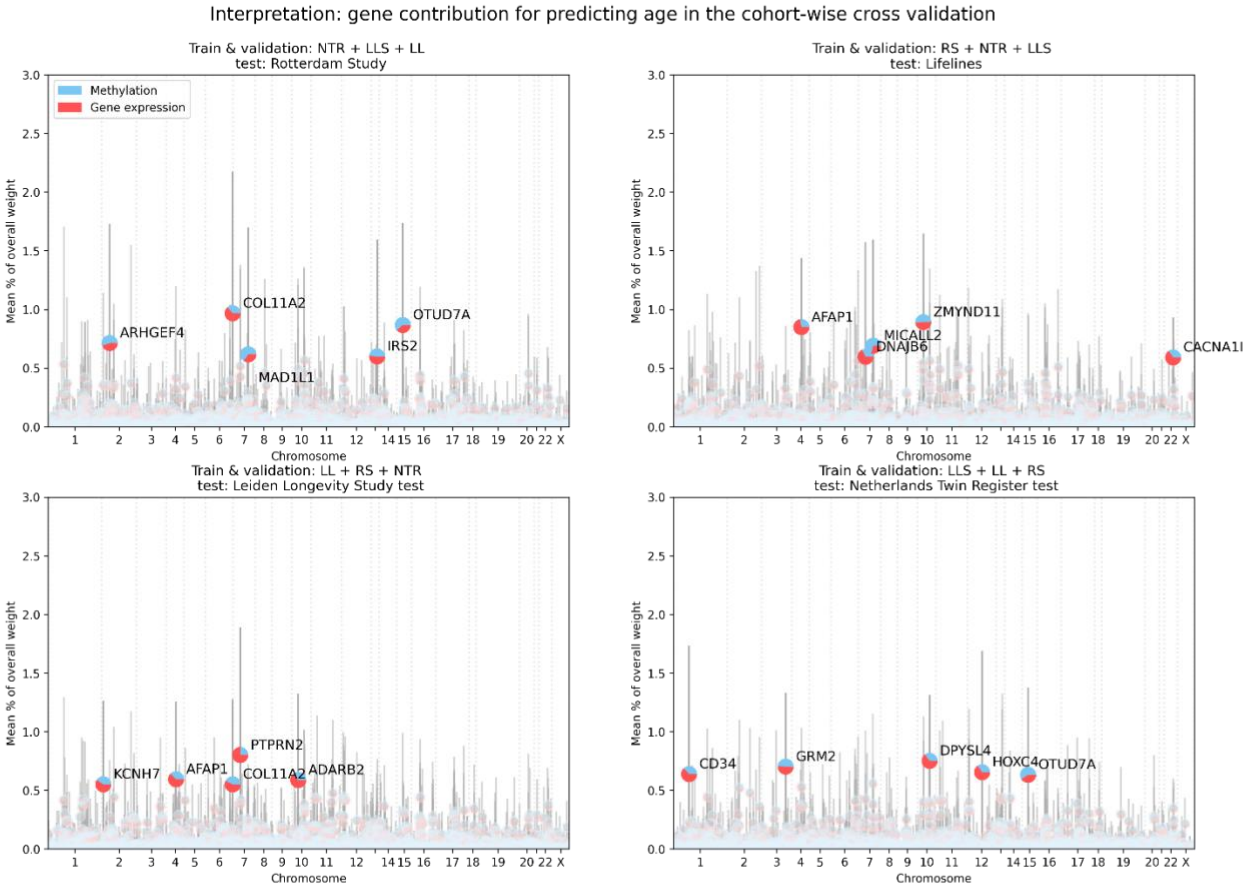

Supplementary Figure 7. Interpratation for networks trained to predict biological age. Mean and standerd deviation over ten runs with different random seed for each fold. Percent of the weights occupied per gene, higher prercentages are more important for predicting biological age.

6. Age: sex specific effects

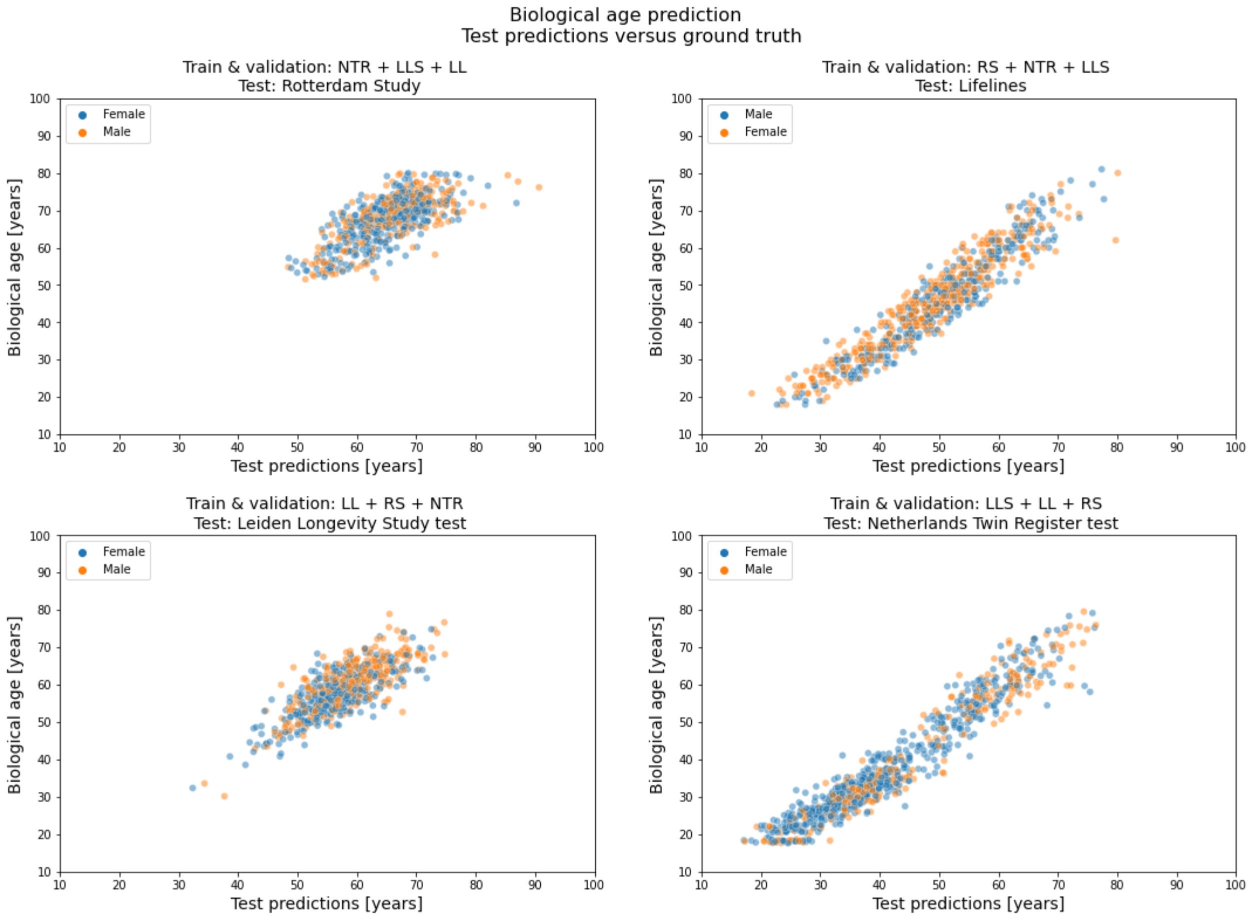

Supplementary Figure 8: Biological age prediction versus the ground truth for each of the test cohorts.

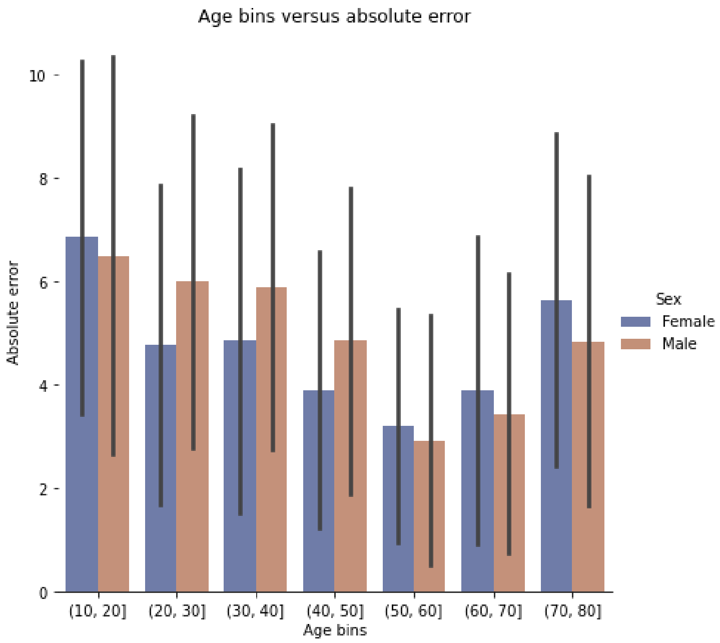

Supplementary Figure 9. Absolute error for biological age prediction binned with a 10-year interval.

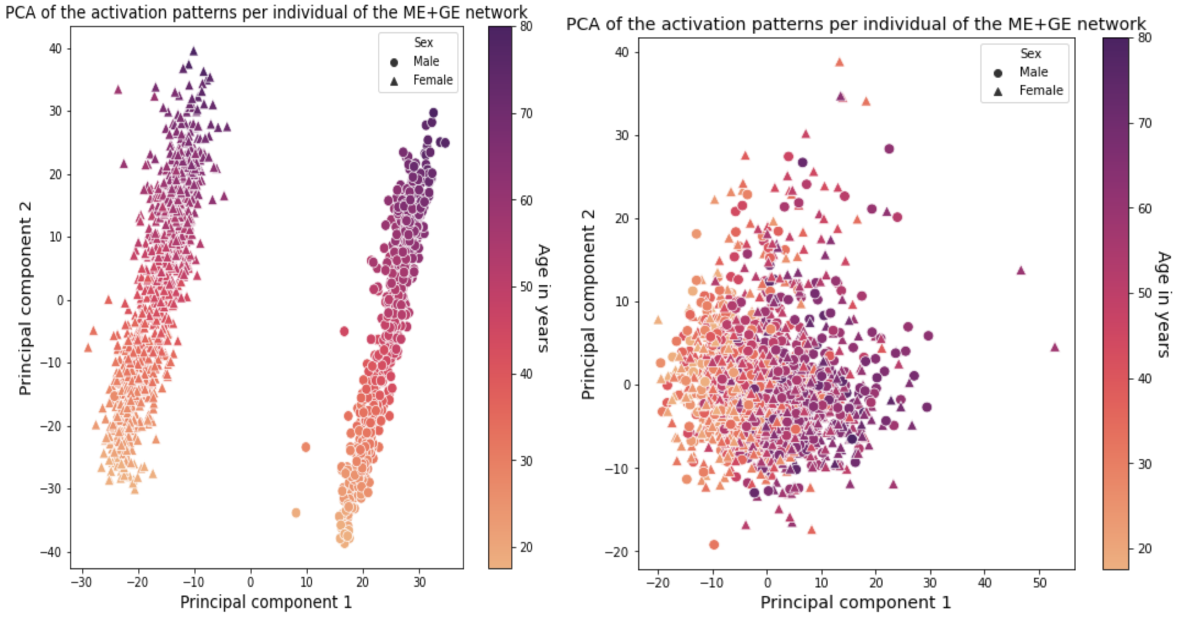

Supplementary Figure 10. PCA of the activation excluding all genes on the X chromosome. Left including genes on the X-chromosome, right excluding genes on the X-chromosome.

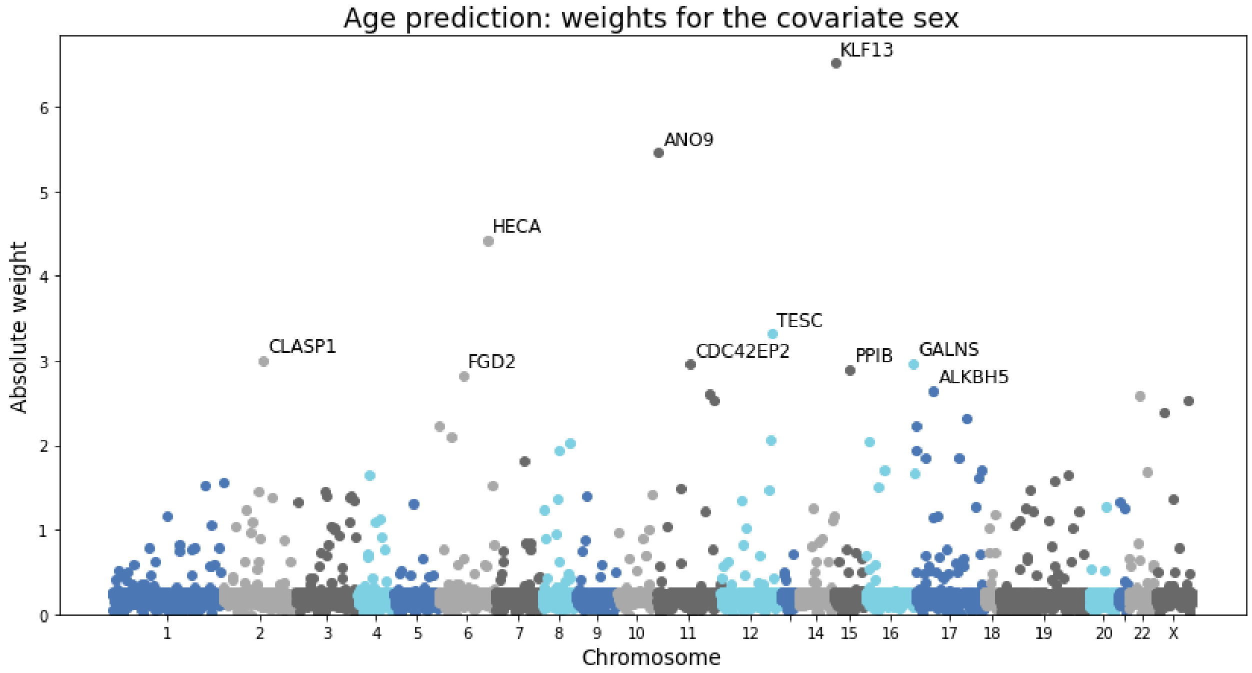

Supplementary Figure 11. Weights for the covariate sex for each gene. Higher weights suggest that there is a larger difference between the sexes.

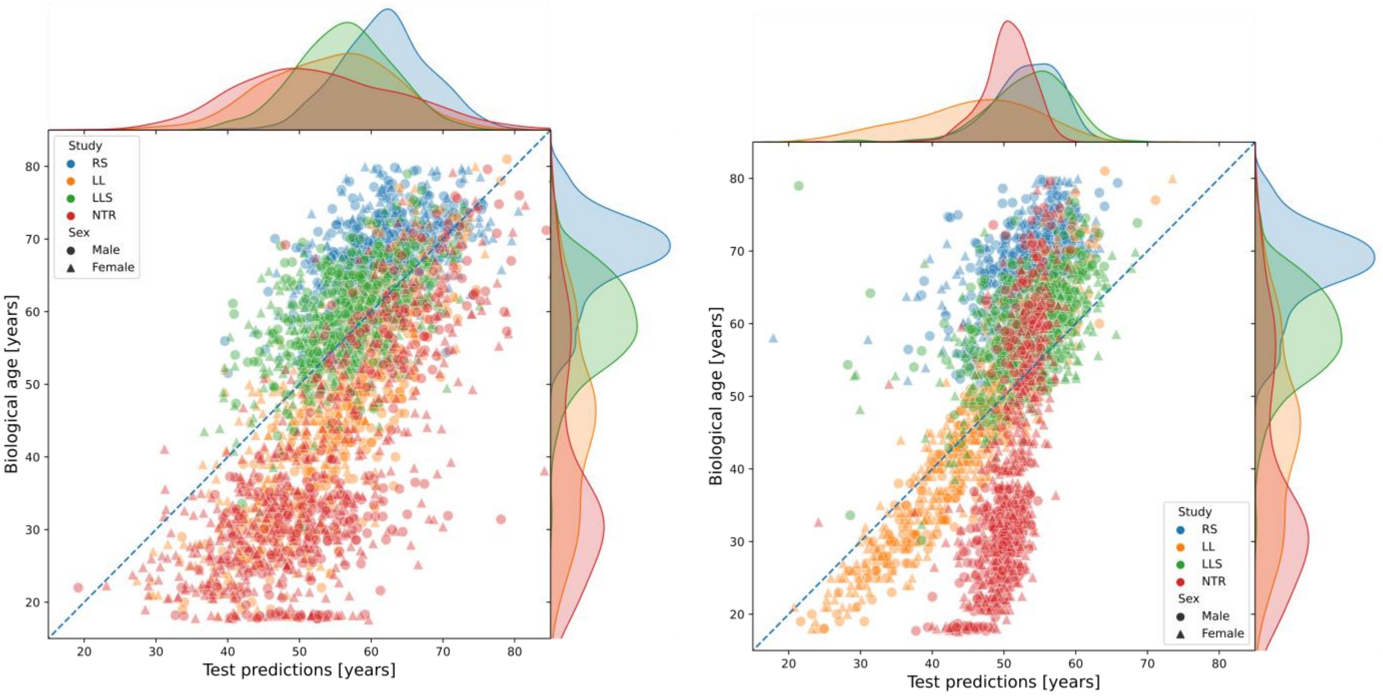

Supplementary Figure 12. Test predictions for each cohort with their corresponding distributions for the GE and ME network, respectively.

7. Age: omic specific effects

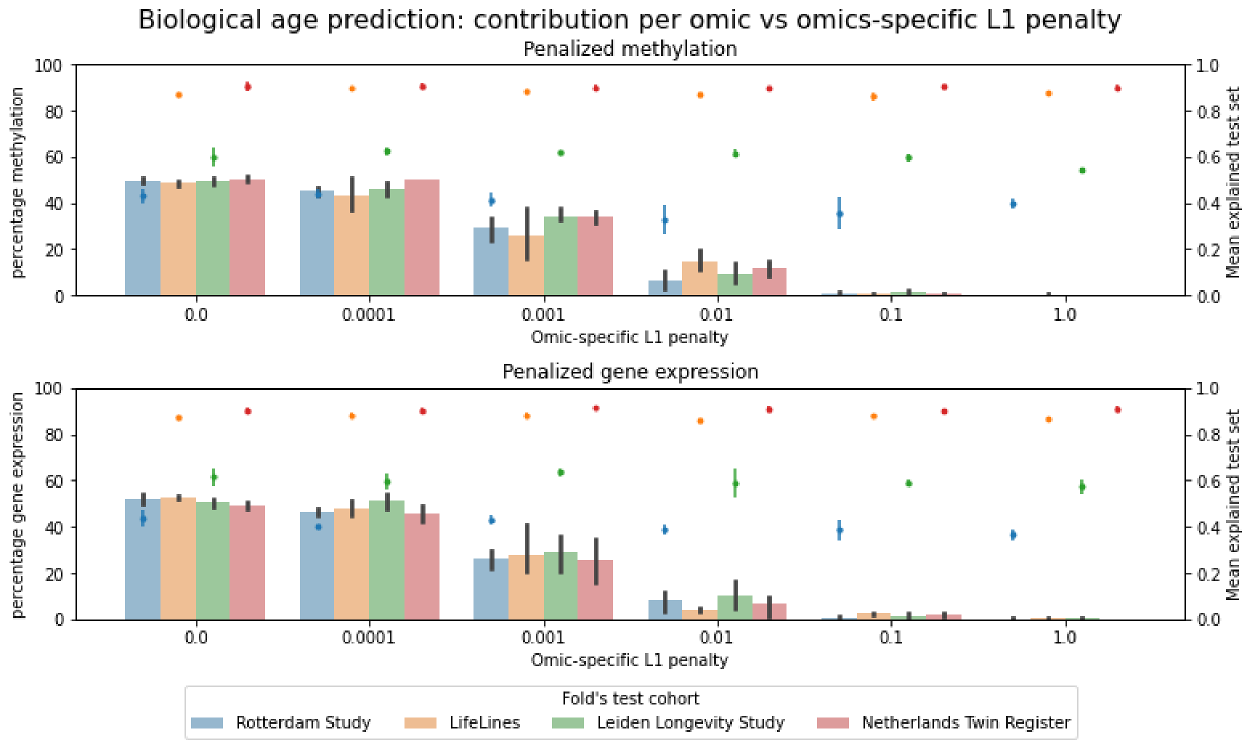

Supplementary Figure 13. Omic specific penalty and its effect on predictive performance and the total percentage of the weight associated with that omic.

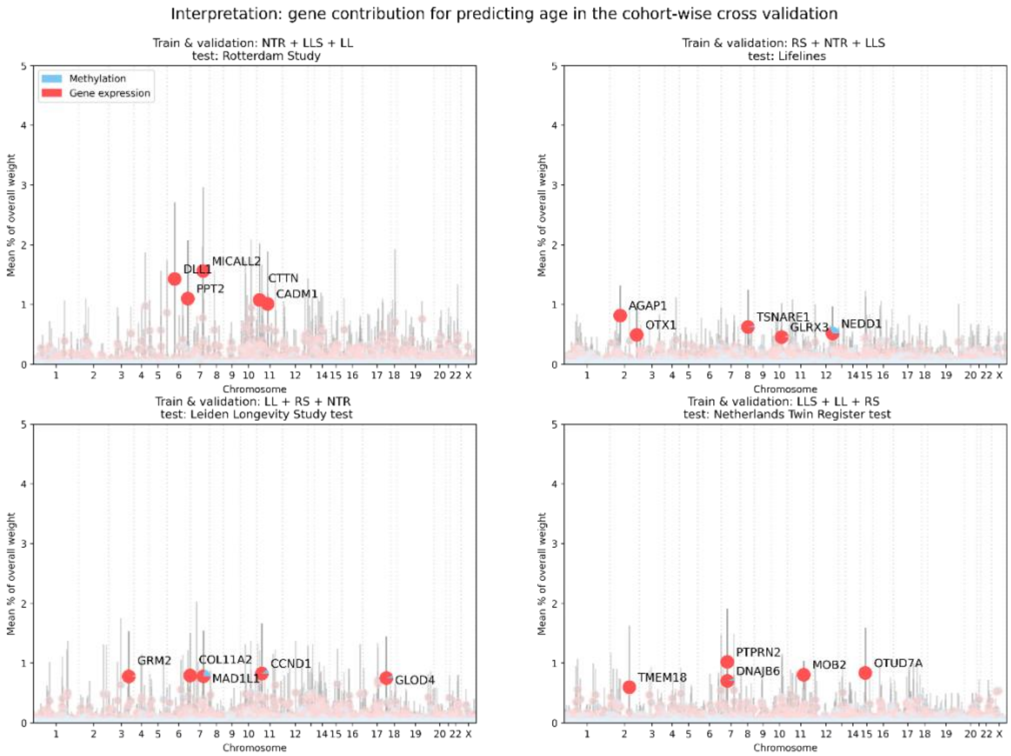

Supplementary Figure 14. Biological age prediction with an omic-specific L1 penalty of 0.01 for the methylation input.

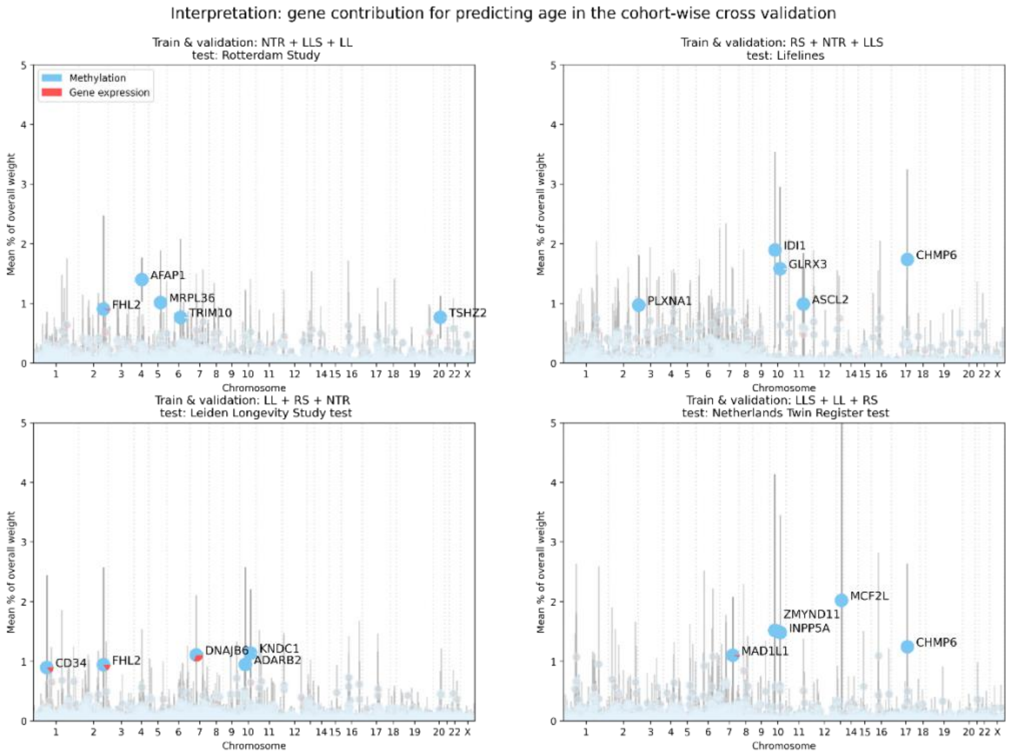

Supplementary Figure 15. Biological age prediction with an omic-specific L1 threshold of 0.01 for gene expression input.

8. Age: pathway importance

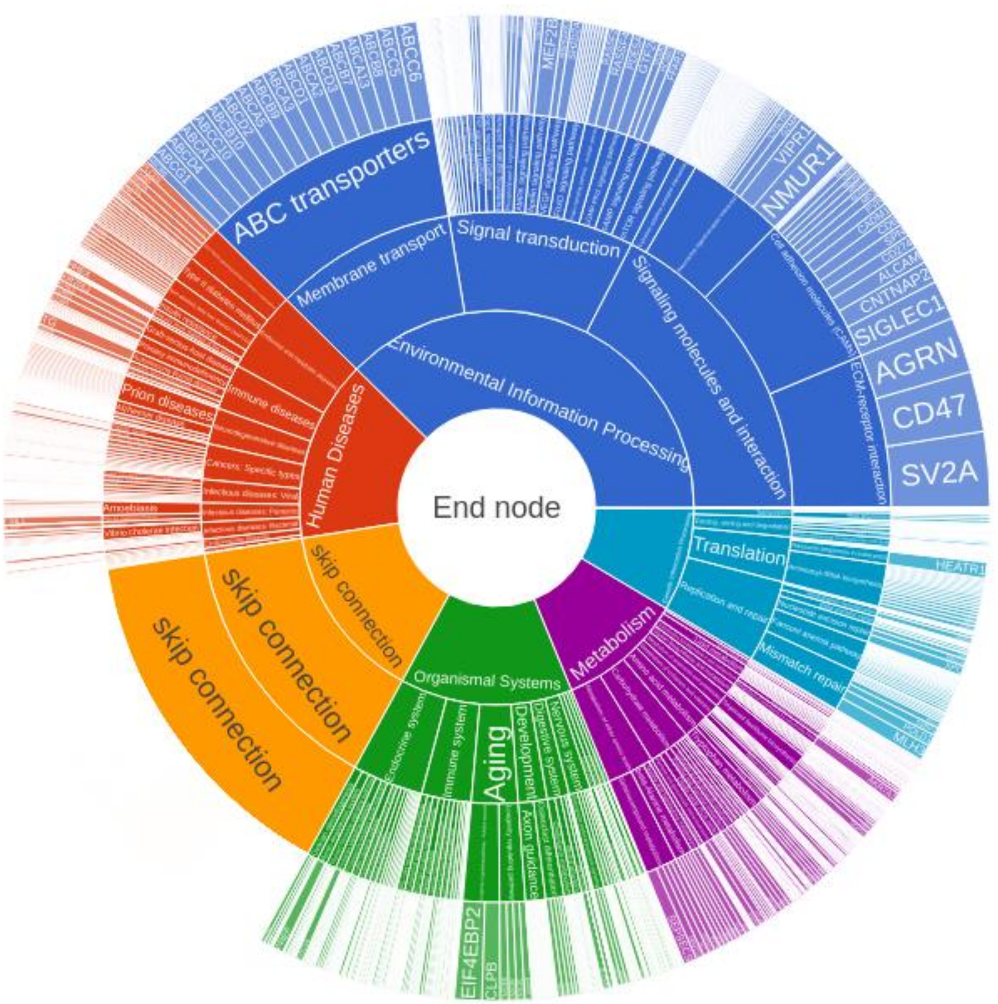

Supplementary Figure 16. Importance of KEGG functional pathways and their corresponding genes for biological age. Skip connections connect each gene right away to the end node to ensure that each gene is connected to the output.

9. LDL prediction

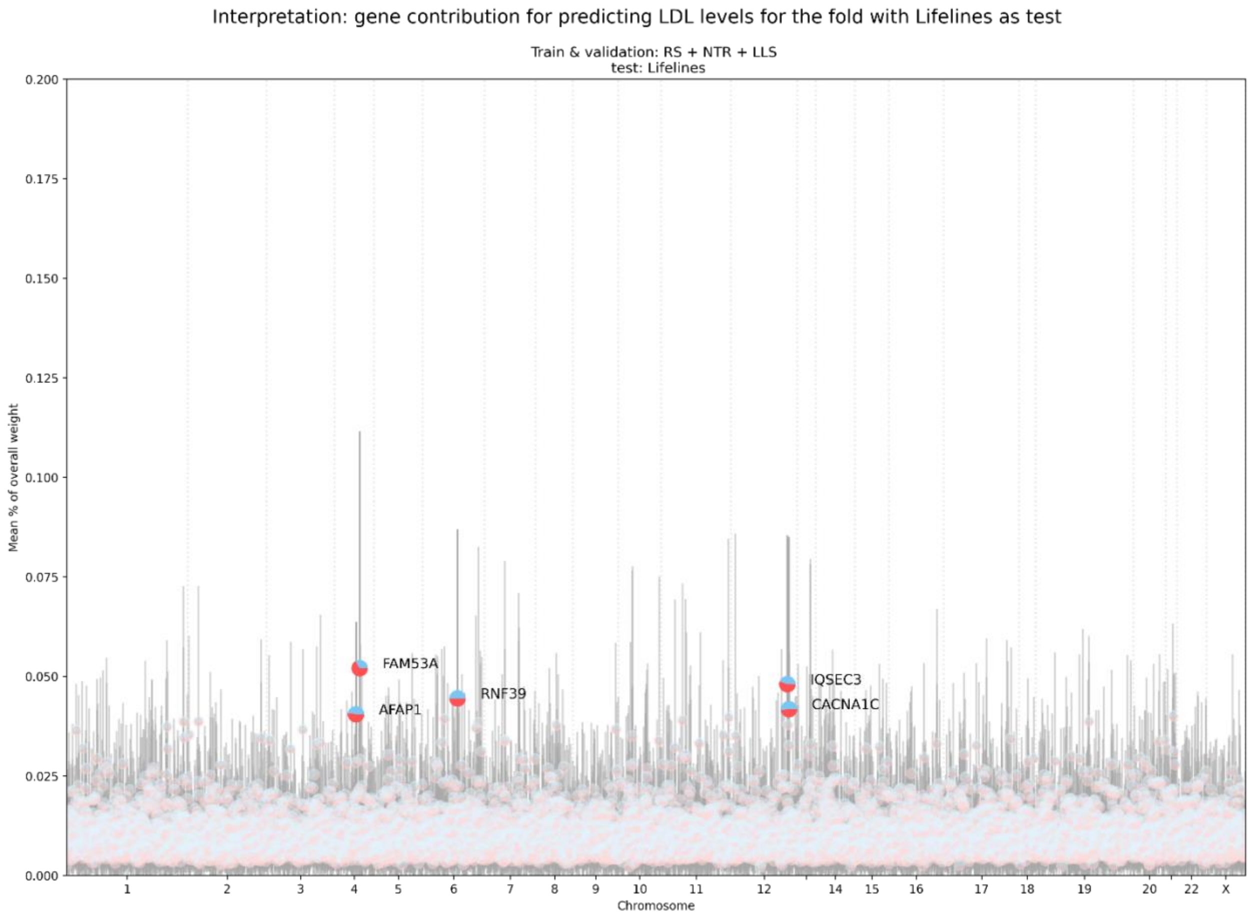

Supplementary Figure 17. Weights for each gene as a percentage of the total weight in the network to predict LDL levels. Only the second fold was predictive and is shown here. Note that the small scale of the y-axis, all weights are close to zero.

References

1. van Hilten, A. *et al.* GenNet framework: interpretable deep learning for predicting phenotypes from genetic data. *Commun. Biol.* **4**, 1–9 (2021).
2. Sigurdsson, A. I. *et al.* Deep integrative models for large-scale human genomics. *Nucleic Acids Res.* **51**, e67–e67 (2023).
3. Lee, H.-J. *et al.* GpNet: Genomic Prediction Network Using Locally Connected Layers in Korean Native Cattle. Preprint at https://doi.org/10.21203/rs.3.rs-622476/v1 (2021).
4. Kassani, P. H., Lu, F., Le Guen, Y., Belloy, M. E. & He, Z. Deep neural networks with controlled variable selection for the identification of putative causal genetic variants. *Nat. Mach. Intell.* **4**, 761–771 (2022).
5. Ngiam, J. *et al.* Tiled convolutional neural networks.
